# Identification of *N*-Oxide-Containing Aromatic Heterocycles as Pharmacophores for Rumen Fermentation Modifiers

**DOI:** 10.3390/metabo9040062

**Published:** 2019-04-02

**Authors:** Carla Bonifacino, Gonzalo Rodríguez, Analía Pérez-Ruchel, José Luis Repetto, Hugo Cerecetto, Cecilia Cajarville, Mercedes González

**Affiliations:** 1 Grupo de Química Medicinal, Laboratorio de Química Orgánica Medicinal, Instituto de Química Biológica, Facultad de Ciencias, Universidad de la República, Iguá 4225, Montevideo 11400, Uruguay; carlaboni9288@hotmail.com (C.B.); gr3093@gmail.com (G.R.); hcerecetto@cin.edu.uy (H.C.); 2 Departamento de Nutrición, Instituto de Producción Animal, Facultad de Veterinaria, Universidad de la República, Ruta 1 km 42,500, San José 80100, Uruguay; anapevet@gmail.com; 3 Área de Radiofarmacia, Centro de Investigaciones Nucleares, Facultad de Ciencias, Universidad de la República, Mataojo 2055, Montevideo 11400, Uruguay; 4 Departamento de Bovinos, Instituto de Produccción Animal, Facultad de Veterinaria, Universidad de la República, Ruta 1 km 42,500, San José 80100, Uruguay; joselorepetto@gmail.com

**Keywords:** rumen fermentation modifiers, propionate, butyrate, *N*-oxide, methylfuroxan, NMR

## Abstract

Different strategies have been used to mitigate greenhouse gas emissions from domesticated ruminants, including the removal of protozoa (defaunation). The objective of the present work was to analyze the potential of different *N*-oxide-containing aromatic heterocycles with known antiprotozoal activity as rumen-gas-abating agents. Nineteen pure compounds, belonging to seven different *N*-oxide chemotypes from our chemo-library were studied together with monensin in an in vitro rumen simulation assay. Fermentation profiles, i.e., gas production, pH, and short carboxylic acid concentrations, were compared to an untreated control at 96 h post inoculation. In our study, we investigated whole-ruminal fluid, with and without compound treatments, by NMR spectroscopy focusing on concentrations of the metabolites acetate, propionate, butyrate, and lactate. From data analysis, three of the compounds from different *N*-oxide chemotypes, including quinoxaline dioxide, benzofuroxan, and methylfuroxan, were able to diminish the production of gases such as monensin with similar gas production lag times for each of them. Additionally, unlike monensin, one methylfuroxan did not decrease the rumen pH during the analyzed incubation time, shifting rumen fermentation to increase the molar concentrations of propionate and butyrate. These facts suggest interesting alternatives as feed supplements to control gas emissions from dairy ruminants.

## 1. Introduction

Ruminants, during enteric fermentation, degrade plant polysaccharides to acetate (Ac), propionate (Prop), butyrate (But), CO_2,_ and CH_4_ via the anaerobic microorganism community in the rumen [1]. The eructated greenhouse gases CO_2_, CH_4_, and H_2_S contribute to global warming [2,3]. Particularly, eructated CH_4_ represents an energy loss to the animals, which can be between 2% and 15% of their gross energy intake [4,5]. The principal methanogens in cattle, belonging to the domain Archaea and the phylum Euryarchaeota, use H_2_ and CO_2_ to generate CH_4_. Methanogens have symbiotic relationships with rumen microorganisms, especially with ruminal protozoa, which involve interspecies hydrogen transfer. Protozoa from the genera *Entodinium*, *Polyplastron*, *Epidinium*, and *Ophryoscolex* have been described to have symbiotic relationships with methanogens [6]. Protozoa compete with amylolytic bacteria for starch, which is fermented into Ac by protozoa while mostly into Prop by amylolytic bacteria. Protozoa are important ruminal H_2_ producers, and the produced H_2_, another rumen gas, is mostly converted into CH_4_ by methanogens situated inside the protozoa or on their external surface. Different gas abatement strategies, usually affecting ruminal microorganisms, have been used with varying success. Some of these gas-mitigating strategies have involved feed-supplementation with lipids, antibiotics, plants or natural products, electron acceptors, and defaunation agents as rumen modifiers [6,7]. Increasing the lipid content of feed is thought to decrease methanogenesis through the inhibition of protozoa, increased production of Prop (hydrogen sink compound), biohydrogenation of unsaturated fatty acids, or toxic effect on cellulolytic bacteria and protozoa [8]. Among antibiotics, the most relevant one is the ionophore monensin (Mon), which is marketed in the USA to increase feed efficiency and weight gain, increase milk production, and decrease milk fat. However, it is banned by the European Union to control antibiotic resistance [6]. Monensin inhibits the growth of gram-negative bacteria and protozoa, which causes a shift towards Prop production in the rumen. In addition, Mon decreases the incidence of subclinical ruminal acidosis by inhibiting the gram-positive bacteria and ciliate protozoa that contribute to lactate (Lac) production [7]. The main natural products, as pure forms or from plants, that have demonstrated the effect of reducing gas, i.e., CH_4_, emissions are essential oils, saponins, and tannins [9]. However, other extracts or isolated natural products have been studied with different results [10,11,12]. Supplementation with nitrates has been also used [7]. The nitrate anion replaces CO_2_ as an electron acceptor and together with H_2_ would produce NH_4_^+^ instead of CH_4_ acting an alternative H_2_ sink in the rumen. The main disadvantage of using a supplementation of nitrates is its great number of secondary effects. Finally, defaunation is the removal of protozoa from the rumen, which inhibits the transfer of H_2_ in the symbiotic relationships between microorganisms and affects the CH_4_ production. For this purpose, different chemical entities have been used, i.e., CuSO_4_, surface-active chemicals, ionophores, triazine, lipids, saponins, and tannins. For almost 20 years, our research group has been working on the development and biological evaluation of different *N*-oxide-containing aromatic heterocycles [13,14,15,16,17,18]. Among the most relevant biological activities, we have found anti-protozoa activity in phenazine dioxide [19], quinoxaline dioxide [20], indazole *N*-oxide [21], benzofuroxan [22,23], benzimidazole dioxide [24], and furoxan derivatives [23,25]. We and others have found that this biological activity could be related to the ability of *N*-oxide moieties as electron acceptors [23,26,27,28,29,30,31,32]. Additionally, some of these *N*-oxides have been studied for their use as substrates for bovine rumen fluid biotransformation [33]; phenazine dioxides, quinoxaline dioxides, indazole *N*-oxides, and benzofuroxans are transformed to reduced products, while assayed furoxan is not biotransformed under the studied conditions.

Taking the above facts into account and with the lack of studies about the use of *N*-oxides as rumen fermentation modifiers, the purpose of this work was to study the effects of compounds belonging to seven *N*-oxide chemotypes on in vitro rumen fermentation.

## 2. Results

### 2.1. Effect of the Studied Compounds on the Total Gas Production

**Figure 1:**
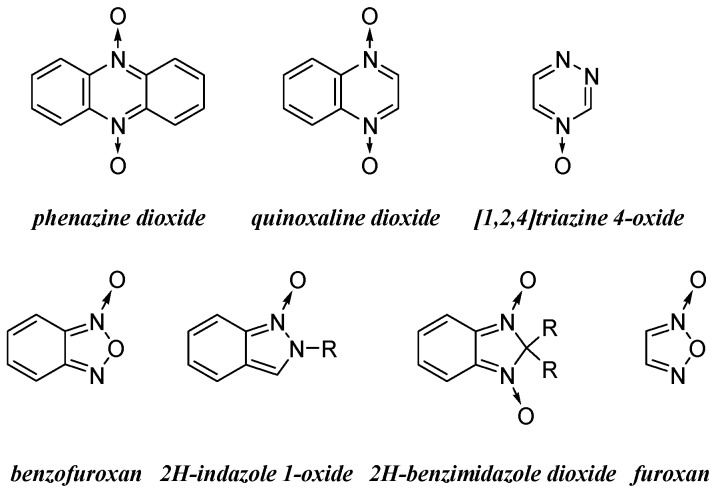
The *N*-oxide chemotypes selected for the study.

**Figure 2:**
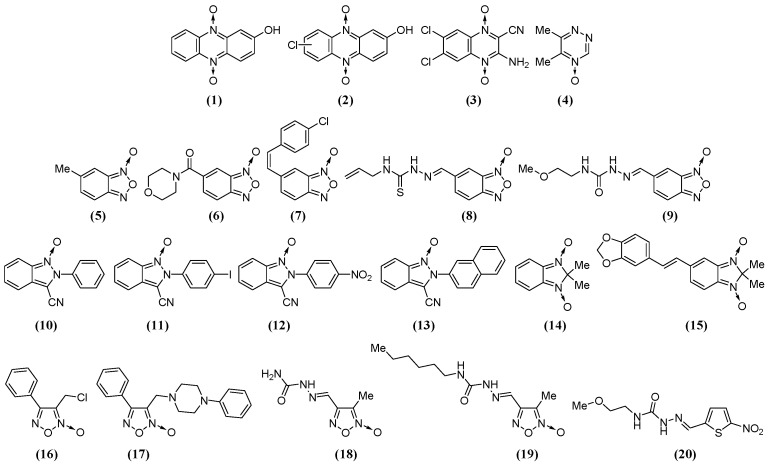
The *N*-oxides and nitrothiophene from our chemo-library studied herein as rumen fermentation modifiers.

In order to identify new pharmacophores for rumen fermentation modifiers, we selected compounds from our chemo-library belonging to different *N*-oxide chemotypes (Figure 1). The selected compounds are shown in Figure 2. After checking their integrity and purity, some of them were synthesized, and for that we followed previously described procedures [17,18,19,21,22,23,24]. The antibiotic ionophore Mon was used in the studies as the positive control. A control without *N*-oxide moiety was also included, i.e., compound **20**, which is structurally related to benzofuroxan **9** porting the same lateral chain.

**Figure 3:**
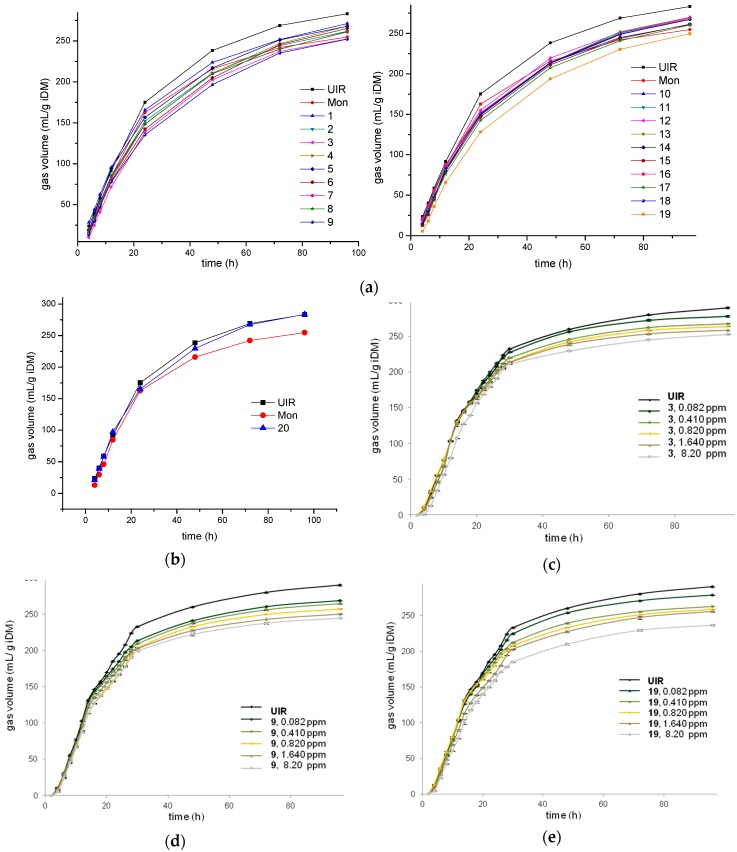
The rumen dynamics of total gas production with or without the studied *N*-oxides at 0.82 ppm (**a**; left: phenazine dioxide, quinoxaline dioxide, triazine 4-oxide, and benzofuroxan derivatives; right: indazole 1-oxide, benzimidazole dioxide, and furoxan derivatives). The error bars were omitted in order to simplify the presentation. The behavior of the selected negative control **20** at 0.82 ppm (**b**), the dose–response curves of gas production for quinoxaline dioxide **3** (**c**), benzofuroxan **9** (**d**) and furoxan **19** (**e**).

The dynamics of the total gas production after the independent inoculation of the nineteen studied *N*-oxides and the controls, i.e., the non-*N*-oxide compound **20** (Figure 2) and the antibiotic ionophore Mon, at 0.82 ppm [34], are shown in Figure 3a. At the end of the experiment, 96 h after inoculation, Mon significantly diminished gas production and increased the lag time, i.e., 254.9 mL/g iDM (mL of gas per gram of incubated dry matter) and 3.1 h, respectively, compared to the untreated incubated rumen (UIR, *p* < 0.05). In this study, UIR was the highest gas producer and had the lowest lag time, 287.3 mL/g iDM and 2.1 h, respectively. Among the *N*-oxides, quinoxaline dioxide **3**, benzofuroxan **9**, and methylfuroxan **19** were the most interesting ones in this assay. Specifically, at the end of the experiment, they significantly decreased the total gas produced (*p* < 0.05, with respect to UIR). For example the “a” values, defined by McDonald [35] as the total gas produced at the time t (according to the model: V = a × (1 − e[*k*d × (t − lag)]), see Materials and Methods Section), were 261.1 mL/g iDM for **3**, 262.5 mL/g iDM for **9**, and 260.7 mL/g iDM for **19**. Additionally, *N*-oxides **3** and **19** displayed similar (*p* < 0.05) lag times compared to that of Mon, 2.9 and 3.4 h, respectively, while benzofuroxan **9** had a similar lag time compared to UIR, i.e., 1.9 h. The constant gas production rates, *k*_d_ (according to McDonald model [35]), for these *N*-oxides were significantly different (*p* < 0.05) from that of Mon and UIR, i.e., 0.035 h^−1^ for **3**, 0.032 h^−1^ for **9** and **19**, 0.046 h^−1^ for Mon, and 0.041 h^−1^ for UIR.

The rest of the studied *N*-oxides also affected the gas production, which was smaller than the UIR production; however, in all cases, they generated more gas than Mon (*p* < 0.05). The non-*N*-oxide control, which was structurally related to benzofuroxan **9** (Figure 2) with the same lateral chain, compound **20**, displayed a behavior not significantly different from that of UIR (Figure 3b).

Gas production dose–response studies were performed for the *N*-oxides with the lowest gas production rates (*k*_d_), i.e., **3**, **9**, and **19** (Figure 3c–e), using 0.082, 0.41, 0.82, 1.64, and 8.20 ppm concentrations. The *N*-oxides linearly decreased gas production with the dose increments. For example, benzofuroxan **9** decreased gas production “a”, with respect to the control, from 9.7% at 0.082 ppm to 17.4% at 8.20 ppm. Similarly, methylfuroxan **19** decreased “a” from 9.9% to 18.3% at the ranged doses. For these three *N*-oxides, the degradation rates and the lag times were not modified with changes in dose.

### 2.2. Effect of the Studied Compounds on Rumen pH

**Figure 4:**
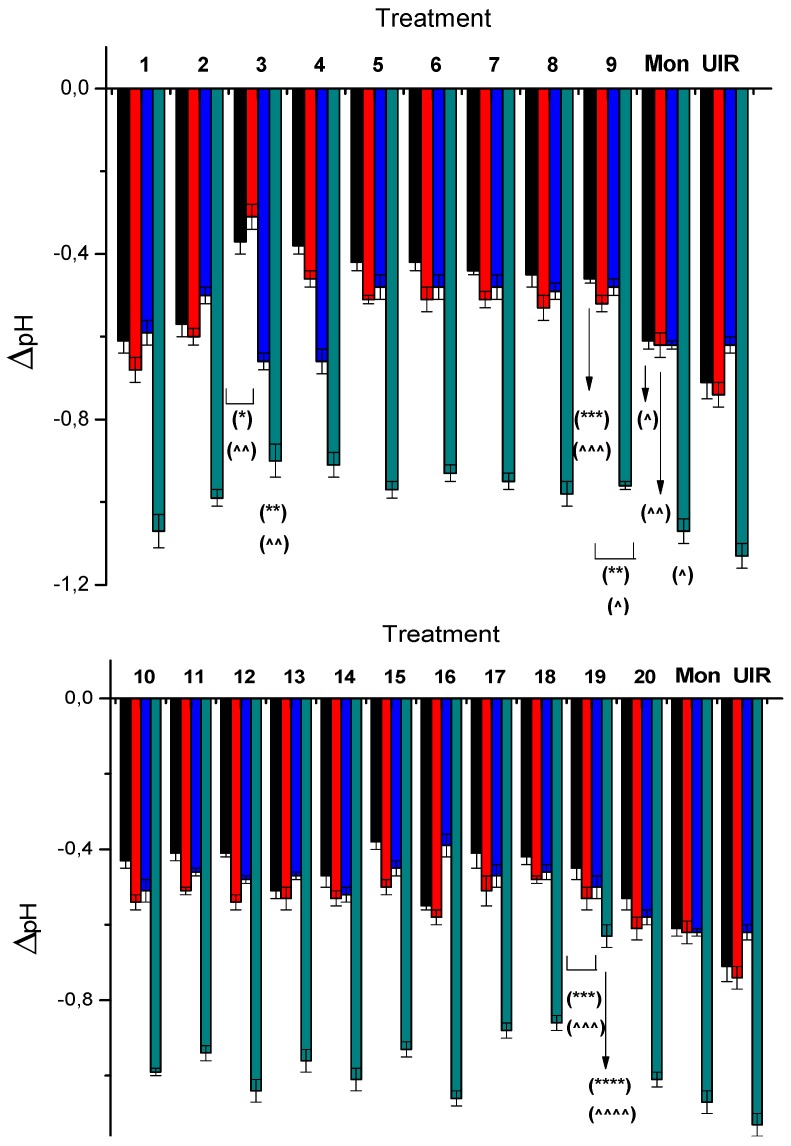
The variation in rumen pH, expressed as ΔpH = pH_initial_ − pH_studied time_ at each checkpoint, with time of the different treatments for the studied compounds at 0.82 ppm (black: 4 h; red: 6 h; blue: 12 h; green: 96 h). (*) *p* = 0.04 with respect to Mon; (**) *p* = 0.03 with respect to Mon; (***) *p* = 0.008 with respect to Mon; (****) *p* = 0.001 with respect to Mon; (^) *p* = 0.04 with respect to UIR; (^^) *p* = 0.008 with respect to UIR; (^^^) *p* = 0.004 with respect to UIR; (^^^^) *p* = 0.001 with respect to UIR.

Under fermentation conditions, the pH of UIR was maintained near 6.6 without significant variations at the first analyzed times (4, 6, and 12 h). The UIR pH fell to 6.1 at the end of the assay (for complete information about values of pH at the different analyzed times see Supplementary Materials, Table S1). The positive control Mon was able to modify this profile at 4, 6, and 96 h post incubation with lower modifications in the pHs (see variations of ΔpH in Figure 4). On the other hand, some *N*-oxide derivatives were able to maintain the change of the rumen pH, ΔpH (Figure 4), lower than both values for Mon and UIR at 4 h post inoculation, i.e., quinoxaline dioxide **3**, triazine 4-oxide **4**, the benzofuroxans (**5**–**9**), indazole 1-oxides **10**–**12**, benzimidazole dioxide **15**, and furoxans **17**–**19**. Quinoxaline dioxide **3** maintained the rumen pH above 6.6 until 6 h post inoculation with a ΔpH significantly lower than both values for Mon and UIR; however, **3** together with triazine 4-oxide **4** produced a lower pH than Mon and UIR after 12 h of incubation, with values (Table S1) decreasing to 6.3. Furoxan **16** was able to maintain the pH nearly constant between 6 and 12 h after inoculation, reaching a pH value of up to 6.7. However, the stability studies showed that it was biotransformed by the rumen; therefore, the chemical identity of the species responsible for the changes in pH was not guaranteed. At the end of the assay, 96 h after inoculation, the pH fell below 6.2 in all of the studied cases, including Mon and UIR, except for methylfuroxan **19**. It was able to maintain the pH at 6.4. Additionally, methylfuroxan **19** was able to produce changes in the pH significantly lower than the corresponding changes for both Mon and UIR at all the studied times (Figure 4).

### 2.3. Effect of the Studied Compounds on the Rumen Short-chain Fatty Acid (SCFA) Composition. Whole Ruminal Fluid ^1^H NMR-Metabolic Profile

Several techniques have been described for determining rumen SCFA composition. One of the most relevant is gas chromatography (GC) [36]. Apart from the requirement equipment, GC involves sample processing, like chemical pre-treatment (with perchloric acid), centrifugation, or filtration, and studied SCFA standard reagents. The use of structural spectroscopy techniques, like NMR, which allow for the unambiguous identification of metabolites has been poorly described [37]. In this work, we performed the analysis of whole-ruminal fluid without pre-treatment by ^1^H NMR spectroscopy focusing our efforts on the analysis of Ace, Prop, But, and Lac concentrations.

The effect of the studied compounds on the rumen SCFA concentration at 0 to 96 h of incubation was studied. These metabolites show characteristic signals in the ^1^H NMR spectrum (Supplementary Materials, Figure S1). The decrease in pH in UIR at the end of the assays was not due to the formation of Lac, as evidenced by the absence of the corresponding ^1^H NMR signal for this SCFA (doublet at 1.316 ppm [38], Figure S1a). The lower pH may have been due to 29.6 mM of Ac, which is the SCFA with the lowest p*K*a (4.76 at 25 °C) among the studied acids. Similarly, at 96 h, Mon-treated rumen fluid did not show Lac, while the concentration of acetate ([Ac]) was lower (*p* < 0.05), i.e., 23.7 mM, compared to UIR; the concentration of propionate ([Prop]), the SCFA with the highest p*K*a (4.87 at 25 °C), was higher (*p* < 0.08), i.e., 15.4 mM, compared to that of UIR, i.e., 13.8 mM (Figure S1a). The mild acidosis at 12 h post inoculation produced by quinoxaline dioxide **3** could have been the result of the higher concentration of Ac (*p* < 0.05), i.e., 25.4 mM, compared to that of UIR, i.e., 22.0 mM, because [Prop] and the concentration of butyrate ([But]) were not significantly different between **3** and UIR and due to the absence of Lac (Figure S1b). On the other hand, methylfuroxan **19**, with an adequate pH value at the end of the assay (Table S1), had the highest [But] and was the SCFA with the intermediate p*K*a (4.83 at 25 °C), i.e., 11.4 mM. The [But] was significantly different (*p* < 0.05) from those of UIR and Mon, i.e., 8.3 mM and 8.5 mM, respectively. Methylfuroxan **19** also had the highest [Prop], i.e., 15.8 mM, which was significantly different (*p* < 0.052) from that of UIR (Figure S1a). The [Ac] at this time, i.e., 30.4 mM, was not significantly different from that of UIR.

For the *N*-oxides with the lowest *k*_d_, i.e., **3**, **9**, and **19,** when the effect on the rumen SCFA concentrations was analyzed, compared to UIR, Mon, and **20** (Table 1), it should be highlighted: (i) Methylfuroxan **19** displayed lower [Ac]/[Prop] and [Ac]/[But] ratios than those of UIR at the end of the assays (*p* < 0.05); (ii) Additionally, methylfuroxan **19** had the lowest [Ac]/[But] ratio, and it was significantly different (*p* < 0.05) from that of Mon; (iii) The *N*-oxides **3** and **9**, such as Mon, also significantly decreased (*p* < 0.05) the [Ac]/[But] ratio at 96 h post inoculation compared to UIR; (iv) Mon rumen SCFA behavior was very different from that of the selected *N*-oxides. It significantly decreased the [Ac]/[Prop] ratio during the assays; (v) On the other hand, the nitrothiophene derivative **20**, which was initially proposed as the negative control, displayed a behavior similar to UIR.

## 3. Discussion

Among the *N*-oxides, three different compounds, i.e., **3**, **9**, and **19**, had the highest gas inhibition effects in the in vitro study, which were close to 10% during all of the treatments and at the same dose as Mon, compared to UIR. Therefore, these compounds could be interesting tools for gas mitigation. Ruminant gas production of CO_2_, CH_4_, and H_2_S represents nearly 80% of greenhouse gas emissions from the livestock sector, 90% of which results from rumen microbial methanogenesis and represents a loss of energy for animal production. Consequently, many studies have been conducted to increase feed efficiency through the manipulation of rumen fermentation. In this sense, we previously observed [39,40] that gas emissions from grazing animals could be reduced by up to 14% by improving pasture quality. The herein studied compounds could be used as an alternative strategy and could be combined with high-quality pastures to reduce these emissions. According to our studies on steer rumen aliquots, quinoxaline dioxide **3** decreased *k*_d_ by 15% with respect to UIR and with a concomitant increase in the lag time of 38%. Quinoxaline dioxides, such as carbadox, olaquindox, and mequindox [41,42], have been used to prevent bacterial infections and to improve animal growth due to their actions against gram-positive and gram-negative bacteria. However, currently they are banned due to health concerns over their (and their metabolites’) toxic effect of oxidative stress [43]. Nevertheless, quinoxaline dioxide **3** is structurally unrelated to these commercial agents because it is a hypoxic-selective cytotoxin [44], ensuring its action at the anaerobic-rumen level and not on the oxygenated tissues of the livestock. On the other hand, benzofuroxan **9** decreased *k*_d_ by 22% with respect to UIR without changes in the lag time. Derivative **9** is structurally different from the rest of the studied benzofuroxans (**5**–**8**, Figure 2), without relevant activities as rumen modifiers, in its benzo-substituent. This substituent is similar to that present in the commercial quinoxaline carbadox, which led us to think that its rumen-modifying activity could be due to this moiety. For that reason, we included compound **20** from our chemo-library, which has this group, in the assays (Figure 2). However, the biological behavior of nitrothiophene **20** confirmed that this moiety by itself is not responsible for the evaluated bioactivities. Finally, methylfuroxan **19** decreased *k*_d_ by 22%, with respect to UIR, with a concomitant increase in the lag time of 62%. It was the best of the studied *N*-oxides as it decreased gas production by nearly 18% at a 8.20 ppm dose (Figure 3e). Comparing derivative **19** to the rest of the furoxans, it is the most lipophilic one due to the hexyl-moiety attached at the semicarbazone group. This could result in a better interaction with the biotarget. Additionally, we previously found furoxans, and specifically melthylfuroxans, were not mutagenic [25,45,46]—a relevant feature when considering methylfuroxan **19** as potential agent to be supplied to production animals.

Normally, the rumen environment has a pH of 6.5. Subacute acidosis has been defined by rumen pH values lower than 5.5–5.8 for several hours a day [47] and acute acidosis by pH values below 5.2 [48]. Ruminal acidosis, initiated by bacteria that produce Lac, results in diarrhea, the production of endotoxins, and cardiovascular and respiratory collapse. Animals respond to this metabolic disorder by reducing their dry matter intake, which reduces milk yield [49]. All of our studied *N*-oxides were able to maintain, at each time-point, the pH changes in the incubated rumen lower than the changes for untreated incubated rumen (UIR). During the incubations, and according to the ^1^H NMR experiments, Lac was not observed in any of the cases. At the end of the assays, all the studied compounds, except methylfuroxan **19**, reached a pH of approximately 6.1, similar to those of UIR and Mon. However, derivative **19** maintained the pH at 6.4 showing the significantly smallest ΔpH at 96 h post inoculation. This could be the result of **19** increasing the molar concentration of Prop, like Mon, i.e., 15.8 mM and 15.4 mM, respectively, and the molar concentration of But, unlike Mon, i.e., 11.4 mM and 8.5 mM, respectively. These results indicate that methylfuroxan **19**, which also modified the gas production profile, is a promising fermentation modifier.

The NMR study of metabolites of the whole ruminal fluid performed herein highlights the relevance of this robust methodological alternative where it is not necessary to process samples or use standards, allowing unequivocal evidence of the chemical entities present in the studied biosystem [37] with shorter analysis times than other traditional methods (acquisition time + processing time: lower than 15 min).

Based on the data obtained from the present study, it could be concluded that some *N*-oxides positively affected the fermentation characteristics. However, methylfuroxan **19** seems to be the most promising among the other identified *N*-oxides in terms of the expected behavior, gas inhibition effects, and controlled pH. Meanwhile, comprehensive in vivo studies with animal hosts need to be undertaken to evaluate the sustainability of **19** supplementation on rumen fermentation modification without detrimental effects on the animal as a whole.

## 4. Materials and Methods

### 4.1. Studied Compounds

The studied compounds (Figure 2) were obtained from our chemo-library after checking their integrity and purity. In some cases, we needed to resynthesize them, and for that process, we followed previously described procedures [17,18,19,21,22,23,24]. Mon was used as the positive control.

### 4.2. Experimental Design

Two experiments were developed. First, all of the compounds were assayed at a fixed dose of 0.82 ppm and were compared with Mon used at the same dose [34]. For this purpose, the compounds were incubated in hermetically sealed bottles to study fermentation as described below. The gas produced for different incubation times was measured in three bottles per compound (replicates). Additionally, two bottles per compound and time (replicates) at incubation times of 0, 4, 6, and 12 h were prepared, simultaneously incubated, and opened to determine the pH and the SCFA content. The entire trial was repeated twice (repetitions).

Second, for the most relevant *N*-oxides (with the lowest gas production rates (*k*_d_)), a dose–response assay was performed at 0.082, 0.41, 0.82, 1.64, and 8.20 ppm and compared with Mon at the same doses, using the same replications and runs as in the first assay.

### 4.3. In Vitro Rumen Assays

Rumen fluid was collected from cannulated steer (Hereford x Holstein male, 440 ± 2 kg body weight), fed a 73% forage (fresh pasture, *Lolium multiflorum*) and 27% concentrate (corn grain and sunflower meal) diet at an intake level of 2.4% of the body weight. Animals had free access to water and were handled according to a procedure approved by the Bioethics Committee of the Veterinary Faculty (Universidad de la República, Montevideo, Uruguay). The rumen fluid was collected from steer approximately 1–2 h after the beginning of the main meal, and it was then filtered through two layers of cheesecloth into a 1 L two-layer, pre-warmed (39 °C) vessel with no remaining air space and purged with deoxygenated CO_2_. The vessels were sealed and transported to the laboratory within 30 min.

The in vitro gas production protocol was carried out as described by Cajarville et al. [50]. Briefly, the rumen fluid (10 mL) was dispensed into pre-warmed 125 mL bottles containing the substrate (0.5 g of a mixture of corn, 0.165 g, and alfalfa, 0.335 g, ground through a 1 mm sieve) and the milieu (40.5 mL). The milieu (purged with deoxygenated CO_2_) was prepared by mixing 38 mL of a basal solution (free of N), 2 mL of a bicarbonate buffer, and 0.5 mL of a reducing agent [51]. All ingredients were mixed under a stream of CO_2_, which flowed into the bottles prior to sealing with butyl rubber stoppers and aluminum crimp seals. Each studied compound dissolved in dimethylsulfoxide (DMSO, 0.5 mL) was dispensed into 1 mL calibrated syringes. Assays only with DMSO (0.5 mL) were included as the UIR. A control without DMSO was also included in the studies to confirm that this amount of DMSO did not affect the systems under study. The mixtures were incubated for 96 h at 39 °C, and each treatment had five replicates, performed in two different runs (Supplementary Materials, Figure S2).

Gas readings were manually taken at 4, 6, 8, 12, 24, 48, 72, and 96 h post-inoculation using a hypodermic syringe (0.8 mm) connected to a digital manometer (SperScientific, 840065, Scottsdale, AZ, USA). After each reading, the gas was vented from the bottle with the syringe. The measured pressure (in psi) was converted into volume (mL), using a calibration equation obtained previously under similar conditions, by connecting the manometer to a three-way stopcock with a syringe to measure gas volume (in mL) and a needle to insert it into the bottles. Readings were corrected for blanks using bottles with buffered rumen fluid without substrate (three per run). The cumulated gas for each incubation time until 96 h was expressed as mL/mass (g) of incubated dry matter (iDM) and fitted to the model proposed by McDonald [35]: V = a × (1 − e[*k*d × (t − lag)]), where “V” (mL/g iDM) is the gas produced at time t, “a” (mL/g iDM) is the total gas produced, “*k*_d_” (h^−1^) is the constant gas production rate, “t” (h) is the time of fermentation, and “lag” (h) is the lag time of gas production.

**Measurement of pH.** At 0, 4, 6, 12, and 96 h time points for the measurement of gas production, some replicate-bottles were opened, and an aliquot of the milieu was taken to immediately measure the pH using a portable digital pH-meter (EV-05991-36, Cole Parmer, Vernon Hills, IL, USA). The probe was calibrated following manufacturer standard protocol. The probe reading was also confirmed with respective standard buffer solutions (pH range: 5.0–8.0) before each measurement to ensure accuracy. The pH changes were expressed as ΔpH defined as pH_initial_ − pH_studied time_ in each checkpoint.

**Stability of the *N*-oxides under the rumen incubation conditions.** All of the studied compounds were incubated under the conditions indicated above for 96 h. Afterwards, the organic compounds were extracted with ethyl acetate, evaporated, and chromatographically analyzed.

### 4.4. NMR Analysis and Data Processing

Firstly, the procedure was validated. Accuracy and precision: 96.8% and 1.2%, respectively, for [Ac]; 98.5% and 0.9%, respectively, for [Prop]; 98.9% and 2.6%, respectively, for [But]. Concentration linearity, between 100 to 1 mM, was checked for each metabolite to yield linear standard plots (*r*^2^ > 0.99).

At 0, 4, 6, 12, and 96 h time points for the measurement of gas production, an aliquot (0.5 mL) was taken from the replicate-bottles to determine the SCFA concentrations. For the NMR spectroscopic studies, each aliquot was centrifuged at 3000× *g* for 10 min. Before measuring, 0.01 mL of dimethylformamide, as internal standard, and 0.09 mL of D_2_O were added to 0.5 mL of the supernatant in 5 mm NMR (Aldrich, St. Louis, MI, USA) sample tubes. One-dimensional ^1^H NMR spectra, ^1^H-^1^H homonuclear, and inverse-detected ^1^H-^13^C correlation experiments were recorded on a Bruker DPX-400 spectrometer at 22.16 °C, operating at a proton NMR frequency of 400.13 MHz. D_2_O was used as the internal lock. Each ^1^H NMR spectrum consisted of 64 scans requiring 10 min and 26 s acquisition time with the following parameters: 0.16 Hz/point, pulse width (PW) = 30° (11.3 ms), and relaxation delay (RD) = 1.5 ms. A pre-saturation sequence was used to suppress the residual H_2_O signal with low power selective irradiation at the H_2_O frequency during the recycle delay. FIDs were Fourier transformed with LB = 0.3 Hz. The resulting spectra were manually phased, baseline corrected, and referenced to internal standard, using Mestre Nova software version 6.0. The chemical displacements used to identify the respective SCFA were previously confirmed by adding each analyzed metabolite to the studied supernatant, as well as by a control solution with 4 mg mL^−1^ of each SCFA in a phosphate buffer, pH = 7.4, using one-dimensional ^1^H NMR spectra, ^1^H-^1^H homonuclear and inverse-detected ^1^H-^13^C correlation experiments. The chemical shifts (δ, ppm) and multiplicity of the SCFA are But, 0.881, triplet; Prop, 1.042, triplet; Lac, 1.316, doublet; Ace, 1.904, singlet. Two controls were used: one with fresh milieu with the corresponding concentration of DMSO used in the samples, and another with UIR. The ^1^H NMR spectra were automatically reduced to ASCII files. Spectral intensities were scaled to internal standard and reduced to integrated regions of equal width (0.04) corresponding to the region of δ 0.0–10.0 by AMIX software. The region of δ 4.85–4.95 ppm was excluded from the analysis because of the residual signal of H_2_O [38,52,53,54,55].

**Statistical Analysis.** The data were analyzed using SAS (SAS Institute Inc., Cary, NC, USA, 2000). The cumulated gas during the incubation times was measured and fitted to the exponential model described, proposed by non-linear regression. The parameters obtained by this regression (a, *k*_d_, and lag), pH, and NMR were compared between compounds using PROC GLM of the SAS package, including the treatment and run in the model. Unless otherwise stated, the means were compared using a Tukey test.

## Figures and Tables

**Table 1:** Effect of the different compounds on the rumen SCFA concentration ratios.

Ratios	[Ac]/[Prop] ^1,2^		[Ac]/[But] ^1^	
Time Post Inoculation	12 h	96 h	12 h	96 h
**3**	2.25 ^(***),(#),(&)^	2.07 ^(***),(&)^	3.41 ^(****),(^),(####),(&),(++)^	2.79 ^(^^^^),(&),(++)^
**9**	2.58 ^(****),(^),(&&),(++++)^	2.05 ^(***),(&)^	4.03 ^(^^^^),(&&&&),(++++)^	2.78 ^(^^^^),(&),(++)^
**19**	2.02 ^(^),(+)^	1.92 ^(^^),(++)^	3.19 ^(****)c^	2.67 ^(*),(^^^^),(++)^
Mon	1.94 ^(^^),(++)^	1.54 ^(^^^),(+++)^	4.01 ^(^^^^),(++++)^	2.81 ^(^^),(+)^
**20**	2.15	2.16	3.14	3.26
UIR	2.21	2.15	3.28	3.56

^1^ (*) *p* = 0.04 with respect to Mon; (***) *p* = 0.008 with respect to Mon; (****) *p* = 0.001 with respect to Mon; (^) *p* = 0.04 with respect to UIR; (^^) *p* = 0.03 with respect to UIR; (^^^) *p* = 0.004 with respect to UIR; (^^^^) *p* = 0.001 with respect to UIR; (#) *p* = 0.03 with respect to **9**; (####) *p* = 0.0009 with respect to **9**; (&) *p* = 0.04 with respect to **19**; (&&) *p* = 0.005 with respect to **19**; (&&&&) *p* < 0.001 with respect to **19**; (+) *p* = 0.04 with respect to **20**; (++) *p* = 0.03 with respect to **20**; (+++) *p* = 0.009 with respect to **20**; (++++) *p* < 0.001 with respect to **20**. ^2^ [Ac]_12_ (mM): 25.4 ± 0.3, for **3**-treatment, 28.2 ± 0.3 for **9**-treatment, 25.3 ± 0.3 for **19**-treatment, 21.3 ± 0.3 for Mon-treatment, 22.6 ± 0.3 for **20**-treatment, 22.0 ± 0.3 for UIR; [Ac]_96_ (mM): 34.6 ± 0. ± 0.4 for **3**-treatment, 32.2 ± 0.4 for **9**-treatment, 30.4 ± 0.4 for **19**-treatment, 23.7 ± 0.3 for Mon-treatment, 27.7 ± 0.4 for **20**-treatment, 29.6 ± 0.4 for UIR; [Prop]_12_ (mM): 11.3 ± 0.1 for **3**-treatment, 10.9 ± 0.1 for **9**-treatment, 12.5 ± 0.1 for **19**-treatment, 11.0 ± 0.1 for Mon-treatment, 10.5 ± 0.1 for **20**-treatment, 9.95± 0.09 for UIR; [Prop]_96_ (mM): 16.7 ± 0.2 for **3**-treatment, 15.7 ± 0.1 for **9**-treatment, 15.8 ± 0.1 for **19**-treatment, 15.4 ± 0.1 for Mon-treatment, 12.8 ± 0.1 for **20**-treatment, 13.8 ± 0.1 for UIR; [But]_12_ (mM): 7.45 ± 0.19 for **3**-treatment, 7.0 ± 0.2 for **9**-treatment, 7.9 ± 0.2 for **19**-treatment, 5.3 ± 0.1 for Mon-treatment, 7.2 ± 0.2 for **20**-treatment, 6.7 ± 0.2 for UIR; [But]_96_ (mM): 12.4 ± 0.3 for **3**-treatment, 11.6 ± 0.3 for **9**-treatment, 11.4 ± 0.3 for **19**-treatment, 8.5 ± 0.2 for Mon-treatment, 8.5 ± 0.2 for **20**-treatment, 8.3 ± 0.2 for UIR.

## Supplementary Materials

The following are available online at https://www.mdpi.com/2218-1989/9/4/62/s1, Figure S1: The selected region of the whole-rumen ^1^H NMR without (**a**, left, t = 96 h; **b**, left, t = 12 h) and with treatment (**a**, center, Mon, t = 96 h; **a**, right, furoxan **19**, t = 96 h; **b**, left, quinoxaline dioxide **3**, t = 12 h). The structures of SCFA are shown as a guide (**a**, left); the signals used for quantifications are marked with full arrows, and those used for identifications are marked with dotted arrows, Figure S2: Schematic experimental protocol. This protocol was applied for compounds **1**–**20**, Mon, and untreated incubated rumen (UIR). The dose-response (gas production) studies were performed similarly (run 1–2 and replicates 1–3) for *N*-oxides **3**, **9**, and **19**, compound **20**, Mon, and UIR, Table S1: Values of pH, during time, in the different rumen-treatments. In green are highlighted some relevant time-points (see text).
