# Supplementary material for: Identification of N-Oxide-Containing Aromatic Heterocycles as Pharmacophores for Rumen Fermentation Modifiers
Source: Metabolites. 2019 Apr 2;9(4):62. doi: 10.3390/metabo9040062 (PMC6523752; doi:10.3390/metabo9040062)
Supplement: Supplementary file 1 [file metabolites-09-00062-s001.pdf]

Identification of *N*-oxide-containing aromatic heterocycles as pharmacophores for rumen fermentation modifiers

Carla Bonifacino, Gonzalo Rodríguez, Analía Pérez-Ruchel, José Luis Repetto, Hugo Cerecetto, Cecilia Cajarville and Mercedes González

[1] Grupo de Química Medicinal, Laboratorio de Química Orgánica Medicinal, Instituto de Química Biológica, Facultad de Ciencias, Universidad de la República, Iguá 4225, 11400 Montevideo, Uruguay
[2] Departamento de Nutrición, Instituto de Producción Animal, Facultad de Veterinaria, Universidad de la República, Ruta 1 km 42,500, San José, Uruguay
[3] Área de Radiofarmacia, Centro de Investigaciones Nucleares, Facultad de Ciencias, Universidad de la República, Mataojo 2055, 11400 Montevideo, Uruguay
[4] Departamento de Bovinos, Instituto de Produccción Animal, Facultad de Veterinaria, Universidad de la República, Ruta 1 km 42,500, San José, Uruguay
* Correspondence: ccajarville@gmail.com; Tel.: +598-43407011

	pH				
**Treatment\time**	**0 h**	**4 h**	**6 h**	**12 h**	**96 h**
**without (UIR)**	7.27 ± 0.01	6.56 ± 0.03	6.53 ± 0.02	6.65 ± 0.01	6.14 ± 0.02
**Mon**	7.20 ± 0.01	6.59 ± 0.01	6.58 ± 0.02	6.58 ± 0.01	6.13 ± 0.02
**1**	7.22 ± 0.02	6.61 ± 0.01	6.54 ± 0.01	6.63 ± 0.01	6.15 ± 0.02
**2**	7.13 ± 0.01	6.56 ± 0.01	6.53 ± 0.01	6.63 ± 0.01	6.14 ± 0.01
**3**	7.00 ± 0.02	6.63 ± 0.01	6.69 ± 0.01	6.34 ± 0.01	6.10 ± 0.02
**4**	6.99 ± 0.01	6.61 ± 0.01	6.53 ± 0.01	6.33 ± 0.02	6.08 ± 0.01
**5**	7.04 ± 0.01	6.62 ± 0.02	6.53 ± 0.01	6.56 ± 0.02	6.07 ± 0.02
**6**	7.04 ± 0.01	6.62 ± 0.01	6.51 ± 0.02	6.56 ± 0.02	6.11 ± 0.01
**7**	7.06 ± 0.01	6.62 ± 0.01	6.55 ± 0.02	6.58 ± 0.02	6.11 ± 0.01
**8**	7.06 ± 0.02	6.61 ± 0.01	6.53 ± 0.02	6.57 ± 0.01	6.08 ± 0.02
**9**	7.05 ± 0.01	6.59 ± 0.01	6.53 ± 0.02	6.57 ± 0.02	6.09 ± 0.01
**10**	7.07 ± 0.02	6.64 ± 0.01	6.53 ± 0.02	6.56 ± 0.03	6.08 ± 0.01
**11**	7.05 ± 0.01	6.64 ± 0.01	6.54 ± 0.01	6.59 ± 0.01	6.10 ± 0.01
**12**	7.10 ± 0.01	6.69 ± 0.01	6.56 ± 0.01	6.62 ± 0.01	6.06 ± 0.02
**13**	7.08 ± 0.01	6.57 ± 0.01	6.55 ± 0.02	6.61 ± 0.01	6.10 ± 0.02
**14**	7.07 ± 0.02	6.60 ± 0.01	6.54 ± 0.01	6.55 ± 0.01	6.06 ± 0.01
**15**	7.01 ± 0.02	6.63 ± 0.01	6.51 ± 0.02	6.56 ± 0.01	6.08 ± 0.01
**16**	7.13 ± 0.01	6.58 ± 0.01	6.55 ± 0.01	6.74 ± 0.01	6.06 ± 0.02
**17**	7.01 ± 0.02	6.60 ± 0.01	6.50 ± 0.02	6.54 ± 0.01	6.13 ± 0.01
**18**	7.00 ± 0.02	6.58 ± 0.01	6.52 ± 0.01	6.54 ± 0.01	6.14 ± 0.01
**19**	7.04 ± 0.01	6.59 ± 0.01	6.51 ± 0.02	6.54 ± 0.01	6.41 ± 0.01
**20**	7.12 ± 0.01	6.59 ± 0.02	6.51 ± 0.02	6.54 ± 0.01	6.11 ± 0.01

Table S1. Values of pH, during time, in the different rumen-treatments. In green are highlighted some relevant time-points (see text).

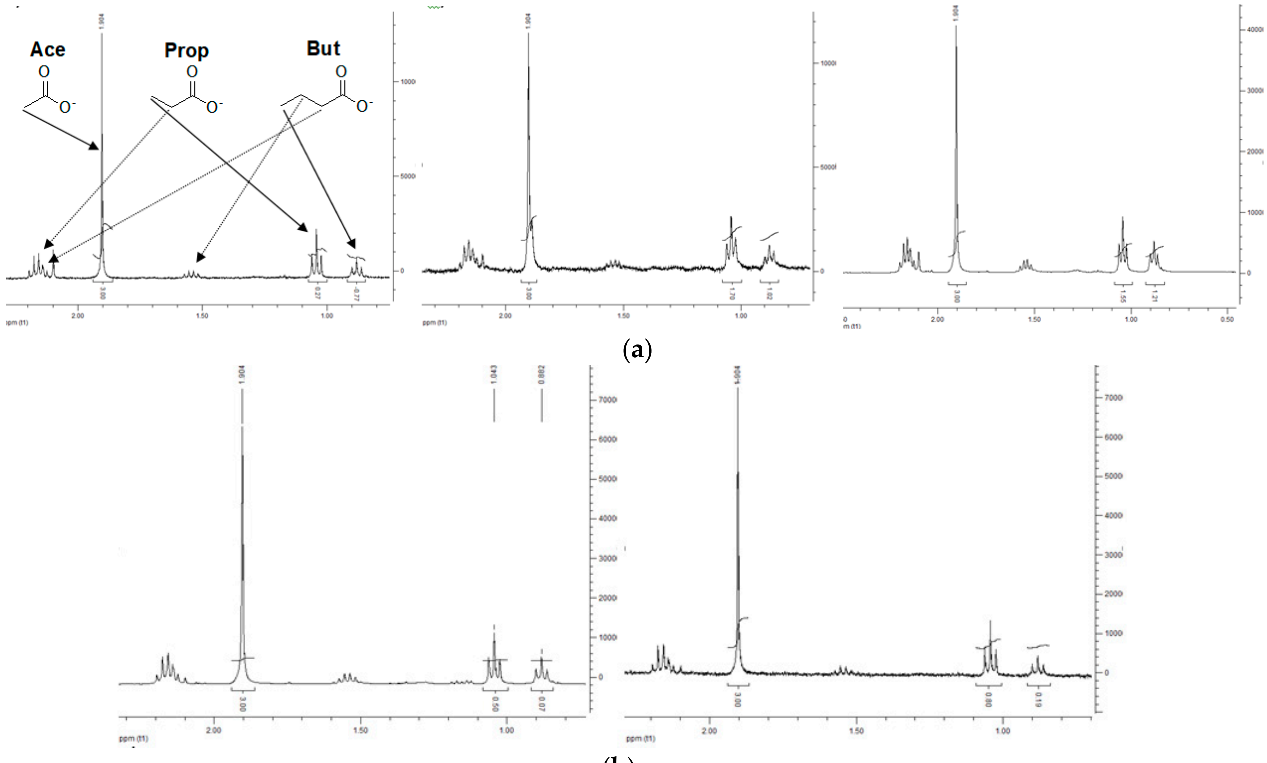

(**a**)

(**b**)

Figure S1. The selected region of the whole-rumen ^{1}H NMR without (**a**, left, t= 96 h; **b**, left, t= 12 h) and with treatment (**a**, center, Mon, t= 96 h; **a**, right, furoxan **19**, t= 96 h; **b**, left, quinoxaline dioxide **3**, t= 12 h). The structures of SCFA are shown as a guide (**a**, left); the signals used for quantifications are marked with full arrows, and those used for identifications are marked with dotted arrows.

replicates

	1	1	1	1	1	1	1	1	1
run 1	2	2	2	2	2	2	2	2	2
	3	3	3	3	3	3	3	3	3

t= 0 h	t= 4 h	t= 6 h	t= 8 h	t= 12 h	t= 24 h	t= 48 h	t= 72 h	t= 96 h
Produced gas is measured of replicates 1-3								

4	4	4	4	4	4	4	4	4
5	5	5	5	5	5	5	5	5

t= 0 h	t= 4 h	t= 6 h		t= 12 h				t= 96 h
Aliquots of each replicate (4 and 5) are taken to measure pH								
Aliquots of each replicate are taken (4 and 5) to measure SCFA								

replicates

	1	1	1	1	1	1	1	1	1
run 2	2	2	2	2	2	2	2	2	2
	3	3	3	3	3	3	3	3	3

t= 0 h	t= 4 h	t= 6 h	t= 8 h	t= 12 h	t= 24 h	t= 48 h	t= 72 h	t= 96 h
Produced gas is measured of replicates 1-3								

4	4	4	4	4	4	4	4	4
5	5	5	5	5	5	5	5	5

t= 0 h	t= 4 h	t= 6 h		t= 12 h				t= 96 h
Aliquots of each replicate (4 and 5) are taken to measure pH								
Aliquots of each replicate are taken (4 and 5) to measure SCFA								

Figure S2. Schematic experimental protocol. This protocol was applied for compounds **1-20**, Mon, and untreated incubated rumen (UIR). The dose-response (gas production) studies were performed similarly (run 1-2 and replicates 1-3) for *N*-oxides **3**, **9**, and **19**, compound **20**, Mon, and UIR.
